# Methotrexate Effectively Controls Ocular Inflammation in Japanese Patients With Non-infectious Uveitis

**DOI:** 10.3389/fmed.2021.732427

**Published:** 2021-11-18

**Authors:** Yosuke Harada, Tomona Hiyama, Yoshiaki Kiuchi

**Affiliations:** Department of Ophthalmology and Visual Science, Graduate School of Biomedical Sciences, Hiroshima University, Hiroshima, Japan

**Keywords:** uveitis, non-infectious uveitis, methotrexate, immunosuppressive therapy, intraocular inflammation, Japanese

## Abstract

This single-center retrospective study investigated the clinical characteristics and efficacy of methotrexate (MTX) for the treatment of non-infectious uveitis for more than 6 months at Hiroshima University, from February 2016 to May 2021. Outcome variables included changes in systemic immunosuppressive treatment and intraocular inflammation. Out of 448 patients with non-infectious uveitis, 35 patients (14 male patients and 21 female patients; 65 eyes) treated with MTX for more than 6 months were analyzed. There were 15 patients with anterior uveitis and 20 with posterior and panuveitis. The mean dose of systemic corticosteroids decreased from 12.1 mg/day at baseline to 1.3 mg/day at 6 months and 0.6 mg at 12 months after starting MTX, and approximately 90% of patients were corticosteroid-free at 12 months. The percentage of eyes with inactive uveitis at 6, 12, and 24 months was 49.2%, 59.6%, and 90.0%, respectively. Mean relapse rate score also significantly decreased from 2.88 at baseline to 0.85 at 12 months (*p* < 0.001). Inflammatory control was achieved with MTX doses of 8–16 mg/week, with a median dose of 12 mg/week. Adverse effects of MTX were observed in 34.3% of patients, and 11.4% required discontinuation; most commonly hepatotoxicity (58.3%), followed by fatigue (25.0%), and hair loss (16.7%). No significant differences were found between the survival curves of patients with anterior uveitis and posterior/panuveitis (Wilcoxon rank-sum test). The percentage of eyes without IOP-lowering eye drops was significantly higher in patients with posterior/panuveitis at 24 months (*p* = 0.001). Our study suggests that MTX is effective in controlling ocular inflammation for Japanese patients with non-infectious uveitis. Relatively high incidence of MTX-related adverse effects in the Japanese population indicates that careful monitoring and dose adjustments are crucial for the long-term use of this therapy.

## Introduction

Uveitis is one of the major causes of vision loss and is estimated to cause approximately 10% of blindness in developed countries (1, 2). Immunosuppressive therapy is the main treatment for non-infectious uveitis; local or systemic corticosteroids are the first-line treatment. However, prolonged corticosteroid use leads to severe ocular (such as cataracts or intraocular elevation) and systemic (such as diabetes mellitus, gastrointestinal complications, or osteoporosis) side effects (3). Thus, corticosteroid-sparing agents should be considered when patients require ongoing treatment and are unable to taper to ≤ 10 mg/day of systemic corticosteroids (4).

Currently, infliximab is approved for Behcet's disease only, and cyclosporine and adalimumab are approved and reported to be efficient for the treatment of non-infectious uveitis in Japan (5–8). Methotrexate (MTX) is a commonly used antimetabolite for ocular inflammation (4, 9–12). Although MTX is administered as a first-line corticosteroid-sparing agent in countries, such as the United States or European countries, there are limited reports from Asian countries, including Japan (13–15). This is because the use of MTX to treat non-infectious uveitis has not yet been established or approved in Japan. Recently, we showed that the administration of MTX at a dose of 8–16 mg/week effectively controlled inflammation in patients with refractory non-infectious scleritis and those who could not tolerate systemic corticosteroids (16). Here, we investigated the efficacy and safety of MTX in the treatment of Japanese patients with non-infectious uveitis.

## Patients and Method

### Study Population

This retrospective study included patients with chronic non-infectious uveitis who were treated with MTX for ≥ 6 months from February 2015 to May 2021 at the uveitis center of Hiroshima University Hospital (Hiroshima, Japan). All patients had active ocular inflammation at the time of MTX initiation despite the use of conventional topical or systemic steroid therapy. The study protocol was approved by the University of Hiroshima Institutional Review Board (protocol number/2122), and the requirement for informed consent was waived.

### Objectives

The primary objectives were to control ocular inflammation and evaluate treatment safety. The secondary objectives included evaluating changes in systemic treatments (e.g., oral corticosteroids) and topical steroids, intraocular pressure (IOP)-lowering therapy, visual acuity improvement, and laser flare photometry results if available.

### Data Collection

For patients treated with MTX, the following data were collected: sex, age at uveitis onset, duration of uveitis, type of uveitis (anterior, intermediate, posterior, or panuveitis), involvement (unilateral or bilateral), diagnosis of uveitis, previous treatment history, surgical history, ophthalmological findings [best-corrected visual acuity, anterior chamber cell grade, vitreous haze grade, retinal/choroidal lesions, and cystoid macular edema (CME)], peripheral anterior synechia, and changes in local and systemic corticosteroid therapy. Adverse events caused by MTX (e.g., hepatotoxicity, fatigue, or hair loss) and the number of patients who required other immunosuppressive treatments were also recorded. Patients who underwent intraocular surgeries within 6 months after the initiation of MTX treatment were excluded from the data analysis. The classification and grading of uveitis were based on the 2005 Standardization of Uveitis Nomenclature criteria (17). For topical steroid therapy, a betamethasone sodium phosphate ophthalmic solution and 0.1% fluorometholone ophthalmic suspension were used for severe and mild anterior uveitis, respectively. Additionally, laser flare photometry values (objective and quantitative measurements of aqueous humor protein levels in the anterior chamber) were analyzed for patients whose flare values were recorded before and after MTX treatment.

Inactive uveitis was defined as an anterior chamber grade ≤ 0.5+, vitreous haze grade ≤ 0.5+, no active retinal/choroidal lesion, and no CME for 3 months. Treatment failure was defined as a two-step increase in the level of inflammation, an increase from grade 3+ to 4+ in the anterior chamber or vitreous haze score, new CME or retinal/choroidal lesion activity, and/or a requirement for corticosteroid rescue therapy. The treatment of patients with bilateral involvement was considered successful only if inactive uveitis was achieved in both eyes. The number of relapses per year was recorded before and after MTX treatment using the following relapse rate scoring system: 0, zero relapses; 1, one relapse; 2, two relapses; 3, three relapses; and 4, ≥ 4 relapses. Steroid-induced ocular hypertension was defined as IOP elevation > 10 mmHg from baseline during topical and systemic corticosteroids therapy.

### Diagnosis

Sarcoidosis and Vogt–Koyanagi–Harada disease were diagnosed in accordance with international diagnostic criteria (18, 19). Sympathetic ophthalmia was diagnosed clinically (20). The diagnosis of multifocal choroiditis was based on the characteristic clinical manifestation of panuveitis and the presence of multiple pigmented chorioretinal lesions in the posterior and mid peripheral retina without any associated systemic or infectious etiologies (21). A patient was diagnosed with retinal vasculitis when a vascular alteration was observed by fluorescein angiography without any associated systemic, infectious, or ocular disorders and associated neoplasms (22).

### MTX Administration

Permission was granted from the Evaluation Committee on Unapproved or Off-labeled Drugs with High Medical Needs at Hiroshima University for unapproved usage of MTX. MTX was initiated when > 10 mg/day systemic corticosteroids were required to control ocular inflammation or patients could not tolerate corticosteroids (e.g., those with steroid-induced ocular hypertension or diabetes mellitus). Patients who were given MTX for systemic diseases, such as rheumatoid arthritis, were excluded from this study. Prior to the administration of MTX, an extensive workup was performed, including chest radiography, exclusion of tuberculosis and syphilis, hepatitis B and C serology, complete blood count, and hepatic and renal function tests. The initial dose was 4–8 mg/week, and it was gradually increased up to 16 mg/week according to the clinical response. The maximum dose of MTX in the present study (16 mg/week) was based on the guidelines for Japanese patients with rheumatoid arthritis (23). Folic acid supplementation was given on the day after MTX administration for patients older than 20 years. To assess MTX-related adverse effects, complete blood count, C-reaction protein, and hepatic and renal function were evaluated at least every 2 months. Regular KL-6 monitoring (every 4 months) and annual chest X-rays were also performed. Tapering of MTX therapy was performed according to the preference of patients who exhibited remission for at least 12 months. MTX was tapered by 2 mg/week every 3–6 months if patients did not experience uveitis recurrence.

### Statistical Analyses

Statistical analyses were performed using Microsoft Excel (Microsoft Corp., Redmond, WA, USA) and JMP version 11 (SAS Institute, Cary, NC, USA). Quantitative variables were described using means, medians, ranges (minimal and maximal values), and counts (percentages). Continuous variables were assessed using the Steel test and Dunnett test. Qualitative variables were analyzed using Fisher's exact test. Comparison of non-parametric data was performed by Mann-Whitney U test. Differences with *p* < 0.05 were considered statistically significant.

## Results

### Demographic and Clinical Characteristics

Among 448 patients with non-infectious uveitis, 50 patients (24 male and 26 female patients) were treated with MTX. Fifteen patients were excluded from the evaluation of efficacy of MTX because it was discontinued within the first 6 months after treatment initiation for the following reasons: adverse effect (nine patients), intraocular surgery (four patients), insufficient inflammation control (one patient), or poor adherence (one patient). In total, 65 eyes in 35 patients (14 male and 21 female) were analyzed for therapeutic outcomes. Their clinical and demographic data are summarized in Table 1. The median age at uveitis onset was 40 years (range, 5–83 years); the median follow-up period was 45 months (range, 10–156 months). The majority of patients had posterior/panuveitis (*n* = 20, 57.1%) and bilateral involvement (*n* = 30, 85.7%). Idiopathic anterior uveitis was the most common diagnosis (*n* = 15, 42.9%). The remaining patients were diagnosed with retinal vasculitis (*n* = 13, 37.1%), biopsy-proven sarcoidosis (*n* = 4, 11.4%), and multifocal choroiditis (*n* = 3, 8.6%). Gonioscopic examination was conducted in 56 out of 65 eyes at presentation; 67% showed peripheral anterior synechia. Most patients (28/35, 80.0%) were treated with corticosteroids before the administration of MTX. Eighteen eyes (22.9%) had a history of intraocular surgeries (cataract surgery, 14 eyes; glaucoma surgery, 5 eyes; vitreous surgery, 2 eyes) before MTX therapy. The median duration of uveitis before MTX treatment was 17 months (range, 3–269 months). The median duration of MTX therapy was 18.5 months (range, 7–48 months).

**Table 1:** Baseline demographic and clinical characteristics of patients with non-infectious uveitis treated with methotrexate.

	**Total**	**%**
Patients	35	
Eyes	65	
Sex		
Male	14	40
Female	21	60
Age at Uveitis Onset, Median in years	40	
Duration of uveitis, Median in months	62	
Type of Uveitis		
Anterior	15	42.9
Intermediate	0	0
Posterior/Pan	20	57.1
Laterality		
Unilateral	5	14.3
Bilateral	30	85.7
Diagnosis		
Idiopathic anterior uveitis	15	42.9
Retinal vasculitis	13	37.1
Sarcoidosis	4	11.4
Multifocal choroiditis	3	8.6

### Control of Ocular Inflammation and Changes in Systemic Treatment

The therapeutic outcomes of MTX therapy for the 35 patients (65 eyes) are summarized in Table 2. No adjustment to systemic treatment was required for non-ocular activity in any patient. The median dose of MTX was 12.0 mg/week (8–16 mg/week). Among the patients who were analyzed, more than 65% needed ≥ 5 mg/day systemic corticosteroids for controlling ocular inflammation (mean dose was 12.1 ± 10.5 mg/day) at baseline, and the remaining proportion could not tolerate corticosteroids due to their adverse effects. After starting MTX, the mean dose of systemic corticosteroids significantly decreased at all evaluation points (3, 6, 12, 18, and 24 months) from baseline (*p* < 0.001). The percentage of patients who no longer required corticosteroids at 6 and 12 months was 68.6 and 88.9%, respectively. A topical steroid was administered in 98.5% of patients at baseline; betamethasone ≥ 2 times daily was used in 80% at baseline and decreased to approximately 40% after 12 months. The proportion of patients who received the less potent topical corticosteroid fluorometholone ≤ 2 times daily was 8.0% at baseline and increased to 34.0% after 12 months (75% changed from betamethasone). Additionally, the proportion of topical steroid treatment-free patients was 1.5% at baseline and increased to 14.9% after 12 months. In contrast, the proportion of patients using IOP-lowering eye drops remained between 34.8 and 46.2% throughout the MTX treatment. Among the eyes treated with IOP-lowering eye drops, glaucoma surgery was performed in 7 patients during MTX therapy. The proportion of eyes with an anterior chamber cell grade and vitreous haze grade ≤ 0.5+ had increased at all evaluation points from base line after starting MTX therapy. The mean best-corrected visual acuity improved from 0.12 logarithm of the minimum angle of resolution at baseline to 0.07, 0.05, and −0.06 at 6, 12, and 24 months, respectively. The proportion of eyes with CME and active retinal/choroidal lesions also decreased after starting MTX. The percentage of eyes (patients) with inactive uveitis at 6, 12, and 24 months was 49.2 (40.0), 59.6 (65.4), and 90.0 (78.5)%, respectively. Additionally, the mean relapse rate score significantly decreased from 2.88 at baseline to 0.85 at 12 months (*p* < 0.001). Treatment failure occurred in 13 patients (37.1%) with a mean survival time of 15.7 months. In six patients (17.1%; five posterior uveitis, one anterior uveitis), after exhibiting remission for at least 12 months, MTX was slowly tapered and discontinued after a median of 23.5 months (range, 21–48 months).

**Table 2:** Treatment outcomes of patients with non-infectious uveitis treated with methotrexate.

	**Base line**	**3 months**	**6 months**	**12 months**	**18 months**	**24 months**
Number of patients (eyes)	35 (65)	35 (65)	35 (65)	26 (47)	17 (30)	14 (23)
Mean dose of methotrexate (mg/week)	0	9.2	10.5	10.6	9.2	9
Systemic corticosteroids						
Mean dose of systemic corticoteroids (mg/day)	12.1	3.1^*^	1.3^*^	0.6^*^	0^*^	0^*^
Percentage of patients with corticosteroids ≤ 5 mg	34.3	72.3	94.3	96.3	100	100
Percentage of corticosteroid-free patients	22.9	45.7	68.6	88.9	100	100
Steroid eye drops						
Percentage of patients with bethamethasone ≥ 2 times daily	80	58.4	58.4	40.3	30	43.4
Percentage of patients with fluorometholone ≤ 2 times daily	8	21.5	24.6	34	40	39.1
None	1.5	9.2	13.3	14.9	26.7	26.1
Percentage of patients with intraocular pressure-lowering eye drops	36.9	46.2	46.2	38.3	36.7	34.8
Mean best corrected visual acuity (logarithm of the minimal angle of resolution)	0.12	0.09	0.07	0.05	−0.04	−0.06
Percentage of eyes with of ≤ 0.5 + anterior chamber cells	32.3	61.5	73.8	74.5	83.3	76.2
Percentage of eyes with ≤ 0.5 + vitreous haze	72.3	86.2	87.7	89.4	100	100
Percentage of eyes with cystic macular edema	90.8	95.4	96.2	95.7	100	100
Percentage of eyes without active retinal/choroidal lesion	44.6	60	70.8	83	96.7	100
Percentage of eyes with inactive uveitis	0	29.2	49.2	59.6	80	90

* *Significant difference observed between baseline and evaluation point (Steel test, p < 0.05)*.

**Figure 1:**
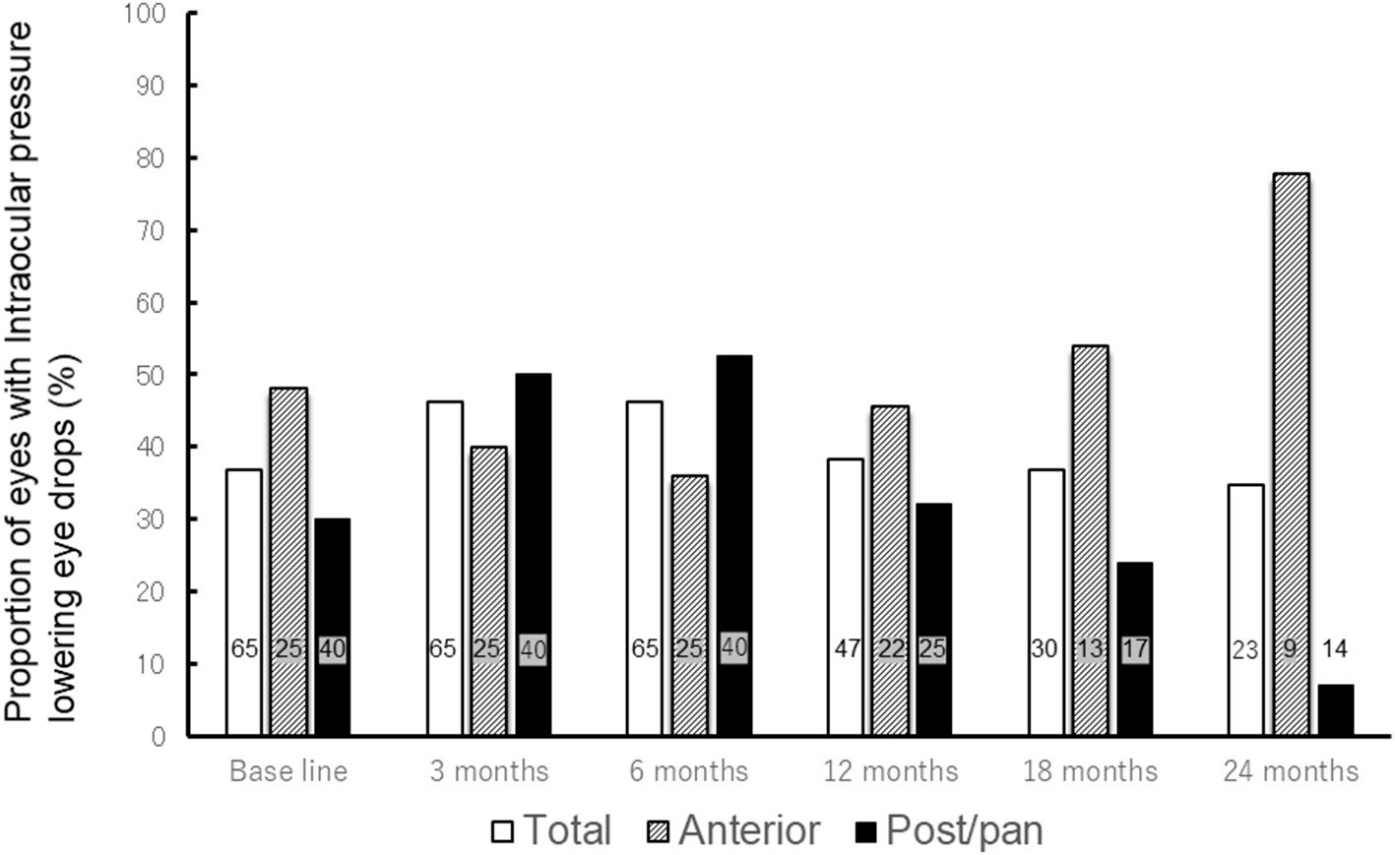
Change in proportion of eyes with intraocular pressure-lowering eye drops after methotrexate therapy. The bar graph shows the change in proportion of eyes with intraocular pressure lowering eye drops after methotrexate treatment (All patients, patients with anterior uveitis, and patients with posterior/panuveitis). The proportion of all eyes with intraocular pressure-lowering eye drops remained between 34.8 and 46.2% throughout methotrexate therapy. While proportion of eyes with intraocular pressure-lowering eye drops decreased from 30.0% at base line to 7.7% at 24 months in patients with posterior/panuveitis, it increased from 48.0% at baseline to 77.8% at 24 months in those with anterior uveitis. The numbers in each bar graph represents the number of eyes using intraocular pressure-lowering eye drops. Total = all patients, Ant = patients with anterior uveitis, Post/pan = patients with posterior/panuveitis.

Treatment outcomes were compared between patients with anterior uveitis (15 patients, 25 eyes) and those with posterior/panuveitis (20 patients, 40 eyes) (Table 3). Although the median follow-up period was 44 months in both groups, the duration of uveitis before starting MTX was significantly longer in patients with anterior uveitis than in those with posterior/panuveitis (median 63.6 months in anterior vs. 13.6 months in posterior/panuveitis, *p* = 0.012). The proportions of patients with peripheral anterior synechia and steroid-induced ocular hypertension were not significantly different between the two groups (*p* = 0.78 and 0.80, respectively). The mean dose of corticosteroids at baseline was 12.6 mg/day and 11.6 mg/day in patients with anterior and posterior/panuveitis, respectively, which decreased to 1.1 mg/day and 0.1 mg/day at 12 months after starting MTX. The percentage of eyes who showed inflammatory control at 12 months after MTX therapy was 59.1% in eyes with anterior uveitis and 60.0% in eyes with posterior/panuveitis, respectively. No significant differences were found between the survival curves of patients with anterior uveitis and posterior/panuveitis according to the Wilcoxon rank-sum test (*p* = 0.70). Five out of 20 (25.0%) patients with posterior/panuveitis discontinued MTX following tapering without recurrence, whereas only one patient with anterior uveitis (6.7%) achieved the discontinuation of therapy. The percentage of eyes without IOP-lowering eye drops was significantly higher in patients with posterior/panuveitis at 24 months (*p* = 0.001) (Figure 1). The number of IOP-lowering eye drops was significantly lower in those with posterior/panuveitis at 18 and 24 months (1.23 in anterior uveitis vs. 0.35 in posterior/panuveitis at 18 months, *p* = 0.046 and 1.89 in anterior uveitis vs. 0.14 in posterior/panuveitis at 24 months, *p* = 0.0007). Among the five patients (eight eyes) with anterior uveitis who started MTX < 1 year after uveitis onset, only one eye was treated with IOP-lowering eye drops before MTX therapy and required no extra IOP-lowering eye drops after the initiation of MTX.

**Table 3:** Treatment outcomes of patients with anterior uveitis and posterior/panuveitis treated with methotrexate.

	**Anterior uveitis**	**Posterior/Panuveitis**
Number of patients, eyes	15 patients, 25 eyes	20 patients, 40 eyes
Median age of onset	39	43
Median follow-up period (months)	44	44
Duration of uveitis before starting methorexate (months)	63.6	12.6^*^
Percentage of eyes with peripheral anterior synechia (%)	68	52.5
Percentage of eyes with steroid induced intraocular pressure elevation (%)	40	45
Mean dose of corticosteroids (mg/day)	12.6	11.6
Baseline		
12 months	1.1	0.1
Percentage of eyes with inactive uveitis at 12 months (%)	59.1	60.1

* *Significant difference observed (Mann-Whitney U test, p < 0.05)*.

### Laser Flare Photometry

Laser flare photometry values were recorded for 17 patients (anterior uveitis: *n* = 9, posterior uveitis: *n* = 7, panuveitis: *n* = 1) before and after MTX therapy. The mean laser flare photometry value was 51.9 ph/ms at baseline and 34.3 ph/ms at 12 months (*p* = 0.93). The patients were then divided into groups according to the duration of uveitis before MTX treatment (Table 4). The mean laser flare photometry value of patients with uveitis for < 15 months (8 patients, 15 eyes) significantly decreased from 27.5 ph/ms at baseline to 10.1 ph/ms at 12 months (*p* = 0.04). On the contrary, the mean laser flare photometry value of patients with uveitis for ≥ 15 months (9 eyes, 14 patients) was 76.5 ph/ms at baseline and remained as high as 60.2 ph/ms at 12 months (*p* = 0.99). Five patients with uveitis for ≥ 15 months (62.5%) underwent intraocular surgery (three glaucoma surgery, one cataract surgery, one vitrectomy), whereas only one patient with uveitis for < 15 months (12.5%) required intraocular surgery (glaucoma surgery). In contrast to the laser flare results, anterior chamber cell grade improved regardless of laser flare values or uveitis duration before MTX administration.

**Table 4:** Change in laser flare photometry values and inflammatory variables according to uveitis duration before methotrexate therapy.

	**Base line**	**12 months**
**Total (17 patients, 29 eyes)**	51.9	34.3
Mean laser flare photometry value (ph/ms)		
Percentage of eyes with anterior chamber cell grade ≤ 0.5+	17.2	72.4^*^
**Patients with uveitis duration < 15 months (8 patients, 15 eyes)**	27.5	10.1^**^
Mean laser flare photometry value (ph/ms)		
Percentage of eyes with anterior chamber cell grade ≤ 0.5+	13.3	66.7^*^
**Patients with uveitis duration ≥ 15 months (9 patients, 14 eyes)**	76.5	60.2
Mean laser flare photometry value (ph/ms)		
Percentage of eyes with anterior chamber cell grade ≤ 0.5+	21.4	78.6^*^

*ph/ms, photons/millisecond*.

* *Significant difference observed (Fisher's test, p < 0.05)*.

** *Significant difference observed (Dunnett test, p < 0.05)*.

### Adverse Effects

Among 35 patients treated with MTX, 12 patients (34.3%) experienced an adverse effect at some point during the follow-up period. These included hepatotoxicity (*n* = 7), fatigue (*n* = 3), hair loss (*n* = 2) Four out of 12 patients discontinued MTX therapy due to adverse effects (11.4%). The median dose of MTX that caused an adverse effect was 10 mg/week (range, 6–16 mg/week).

## Discussion

Our findings indicated that MTX is effective for controlling inflammation and reducing the relapse rate in Japanese patients with non-infectious uveitis. Ocular inflammation was controlled in approximately 65 and 80% of patients at 12 and 24 months, respectively. The mean systemic corticosteroid dose decreased significantly after MTX therapy. More than 85% of patients no longer required steroid treatment at 12 months after MTX initiation. A median dose of 12 mg/week was required to control ocular inflammation. No improvement was observed in the number of IOP-lowering eye drops before and after MTX therapy, especially in patients with anterior uveitis. This study also suggested a benefit of early MTX administration in reducing the risk of ocular complications due to elevation of laser flare values, owing to irreversible disruption of blood-aqueous barrier.

MTX is one of the most commonly used immunosuppressive agents for non-infectious uveitis and several reports have demonstrated the efficacy of MTX for non-infectious uveitis; most of these studies were reported in patients from the United States, Europe, South America, Australia, or India (9–12, 24). The median dosage of MTX that most uveitis specialists in the American Uveitis Society administer is 20 mg/week (25). However, considerably higher incidence of some serious adverse events of MTX (such as pneumocystis pneumonia, lymphoproliferative disorders, and hepatotoxicity) have been reported among Japanese patients than in those from other countries (23). For Japanese patients with rheumatoid arthritis, the maximum dose of MTX is limited to 16 mg/week and Japan College of Rheumatology guidelines recommends the dose escalation up to 10-12 mg/week, because it has been reported that these doses can maintain the adequate concentration of MTX to suppress inflammation with limited adverse effects on liver function (23, 26). Only few studies from East Asian countries have demonstrated the use of MTX to treat non-infectious uveitis; lower doses of MTX ≤ 8 mg/week was administered in some of the reports (13–16, 27). In our study, inflammatory control was achieved with MTX doses of 8–16 mg/week, with a median dose of 12 mg/week, which is similar to the dose used for Japanese patients with RA. These results indicate that although it is still lower than the MTX which is usually prescribed in Western countries, higher doses of MTX than previously reported in East Asian populations may be required to control ocular inflammation. Despite the small number of patients, we believe it is worthwhile reporting our clinical experience using MTX for the treatment of non-infectious uveitis. Additional prospective studies from East Asia are needed to determine the adequate dose of MTX for ocular inflammation.

According to previous studies from Western countries, the incidence of MTX-related adverse events varied greatly; the percentage of discontinuation caused by adverse events ranged from 2.8 to 26% (10, 11, 24, 28). In our study, 34.3% of patients experienced adverse effects, and 11.4% had to discontinue MTX. Although there were no serious adverse events, such as lymphoproliferative disease, close monitoring during MTX therapy for non-infectious uveitis is essential.

The difference in MTX treatment efficacy among various types of uveitis remains controversial. Gangaputra et al. (10) reported that posterior or panuveitis responded less to MTX therapy than other types of uveitis, whereas Rathinam et al. (12) showed a better response of posterior or panuveitis than anterior and intermediate uveitis. Our results were consistent with the latter report; patients with posterior and panuveitis required a lower dose of MTX and achieved better efficacy than those with anterior uveitis. Additionally, the rate of MTX discontinuation due to remission was higher in patients with posterior/panuveitis. There are several reasons to explain these results. First, the duration between uveitis onset and MTX therapy was longer in patients with anterior uveitis than in those with posterior/panuveitis. Most patients were treated solely with local corticosteroids for a longer period before being referred to our uveitis center. In fact, a longer duration of chronic anterior uveitis is reported to be one of the factors associated with failure to achieve remission (29). Second, patients with anterior uveitis included in this study may have had more severe forms of inflammation at baseline than patients with posterior/panuveitis. Further studies with a larger number of patients are needed to address this question.

Ocular hypertension and glaucoma are common and one of the serious complications of uveitis (30–33). Reduced local and systemic corticosteroid use and rates of ocular hypertension may be obtained from the introduction of immunosuppressive therapy (31). In this study, despite the reduction in local and systemic steroid therapies, there was no decrease in the number of IOP-lowering eye drops, especially for patients with anterior uveitis. The longer duration of active inflammation before MTX initiation, which causes irreversible structural changes to the trabecular meshwork, may have contributed to this result. In fact, only one out of eight eyes was treated with IOP-lowering eye drops before MTX therapy, and no eye drops were added after treatment in patients with anterior uveitis for < 12 months before MTX initiation.

Previously, it has been reported that high laser flare values are associated with ocular complications, regardless of the presence of anterior chamber cell grade (6, 34–36). In our study, patients who received early MTX treatment achieved reduced laser flare photometry values and surgical requirements for ocular complications. The findings support the need for MTX administration before the irreversible disruption of the blood-aqueous barrier due to chronic inflammation.

The current study had several limitations. First, it was a retrospective study. Second, although the number of patients is larger than previous reports from East Asia, it is still limited. Our study would have been strengthened by including multiple Japanese institutions to determine the efficacy, safety, and adequate dose of MTX in the Japanese population. Third, a longer follow-up period is necessary for further assessment of MTX efficacy and adverse effects. Finally, we collected data from all eyes affected with uveitis (both eyes for some patients) to prevent a potential loss of information, which can affect the independence of the sample and the statistical analysis about clinical findings of each eye.

In conclusion, this study demonstrated that MTX treatment is effective in controlling ocular inflammation for Japanese patients with non-infectious uveitis. MTX dose similar to that used for Japanese patients with rheumatoid arthritis was sufficient to control ocular inflammation. Although MTX had a corticosteroid-sparing effect both topically and systemically, it did not decrease the use of IOP-lowering eye drops for patients with ocular hypertension. Relatively high incidence of MTX-related adverse effects in the Japanese population indicates that careful monitoring and dose adjustments are crucial for the long-term use of this therapy.

## Data Availability Statement

The data that support the findings of this study are available on request from the corresponding author, YH. The data are not publicly available due to their containing information that could compromise the privacy of research participants.

## Ethics Statement

The studies involving human participants were reviewed and approved by University of Hiroshima Institutional Review Board. Written informed consent to participate in this study was provided by the participant's legal guardian/next of kin.

## Author Contributions

YH and TH contributed to conception and design of the study, organized the database, performed the statistical analysis, and wrote the first draft of the manuscript. All authors contributed to manuscript revision, read, and approved the submitted version.

## Conflict of Interest

The authors declare that the research was conducted in the absence of any commercial or financial relationships that could be construed as a potential conflict of interest.

## Publisher's Note

All claims expressed in this article are solely those of the authors and do not necessarily represent those of their affiliated organizations, or those of the publisher, the editors and the reviewers. Any product that may be evaluated in this article, or claim that may be made by its manufacturer, is not guaranteed or endorsed by the publisher.
